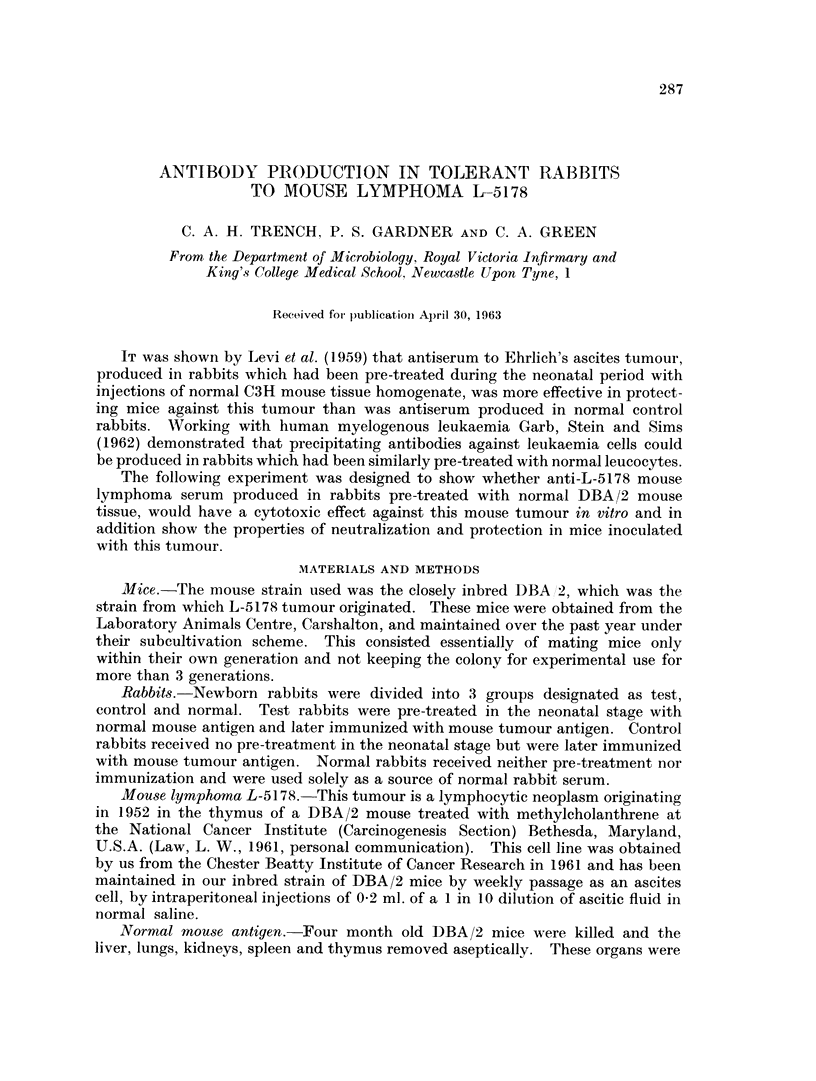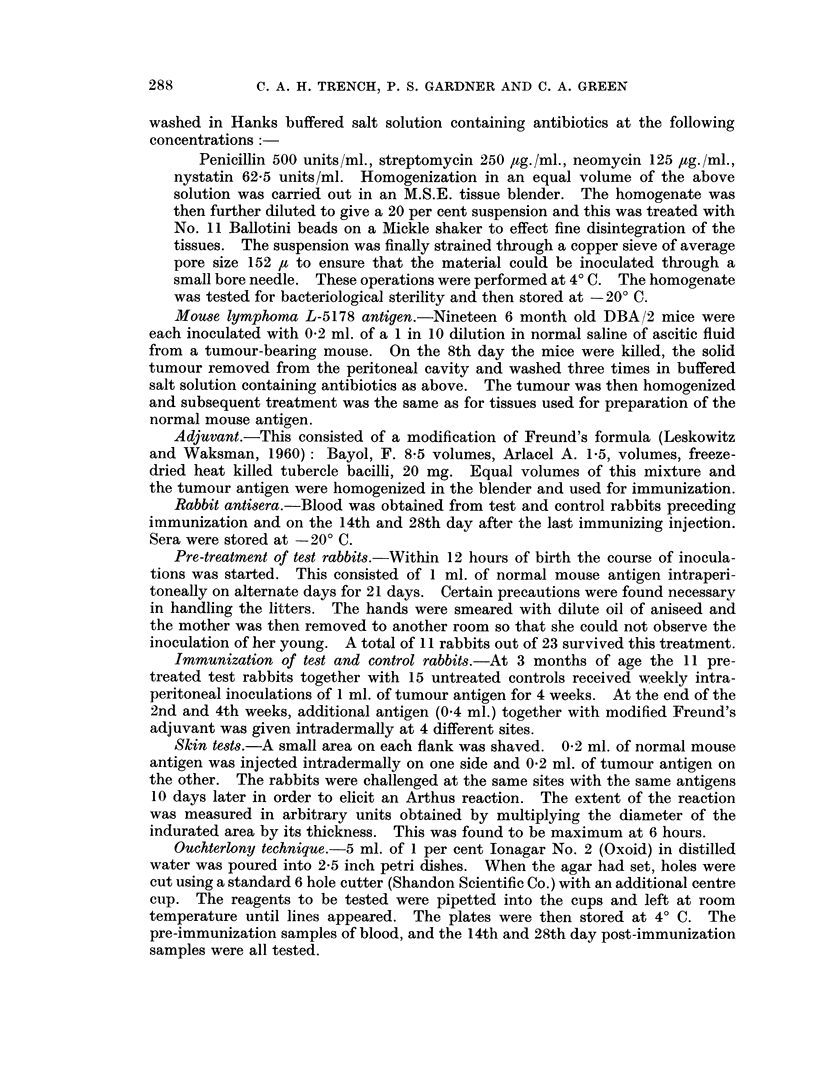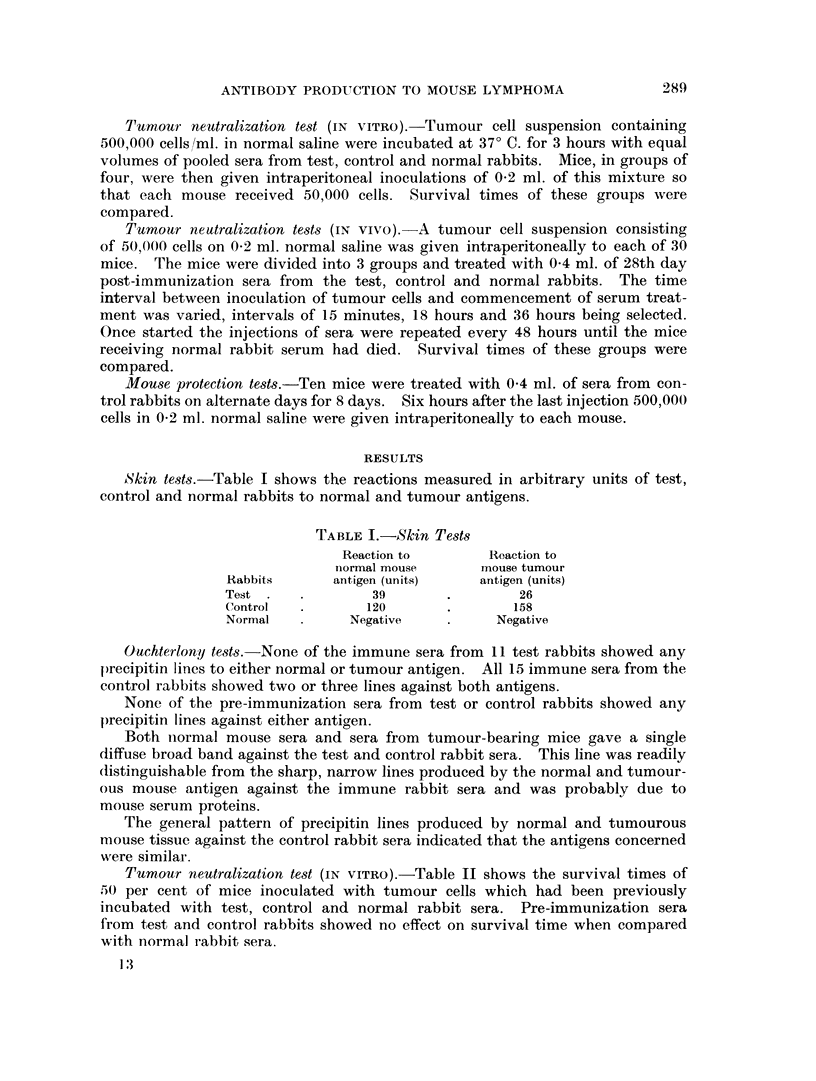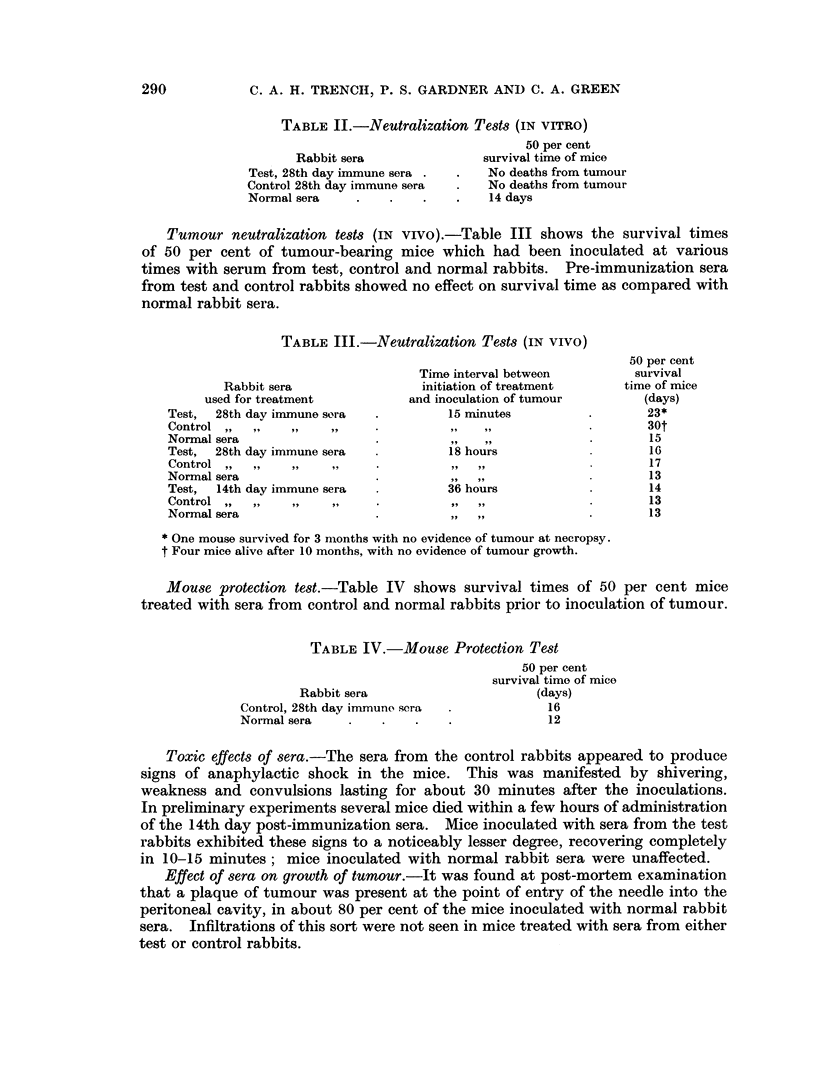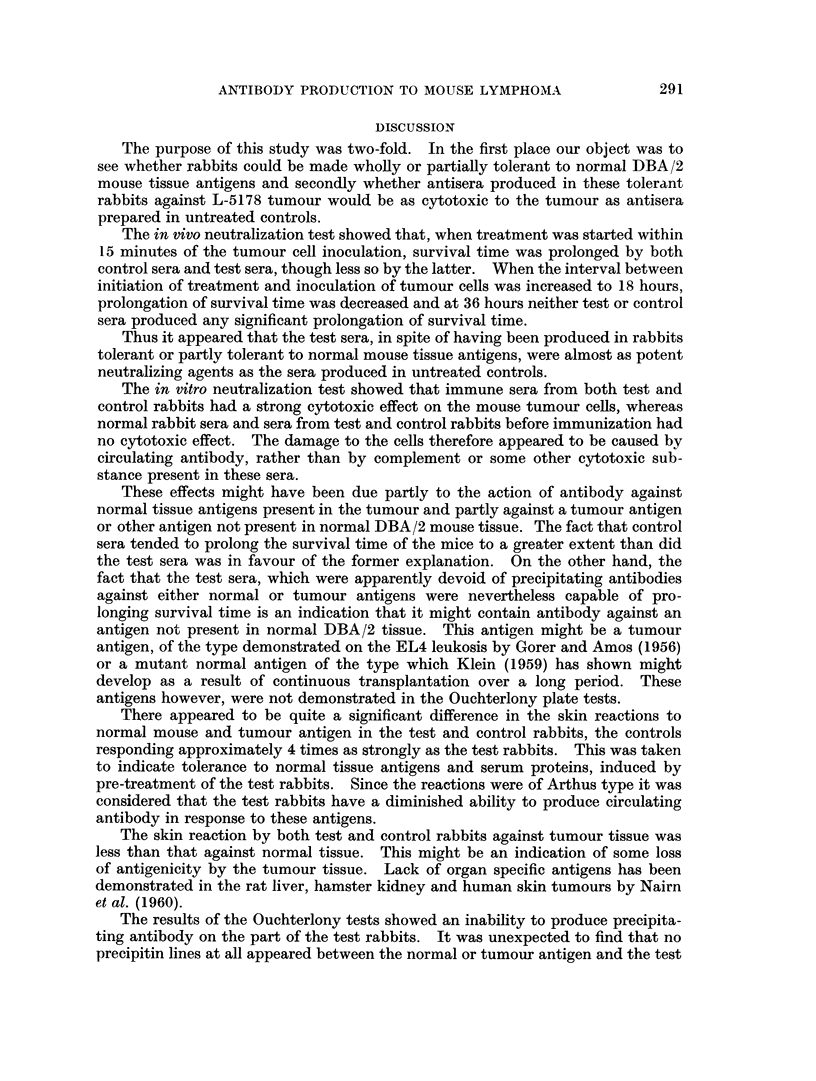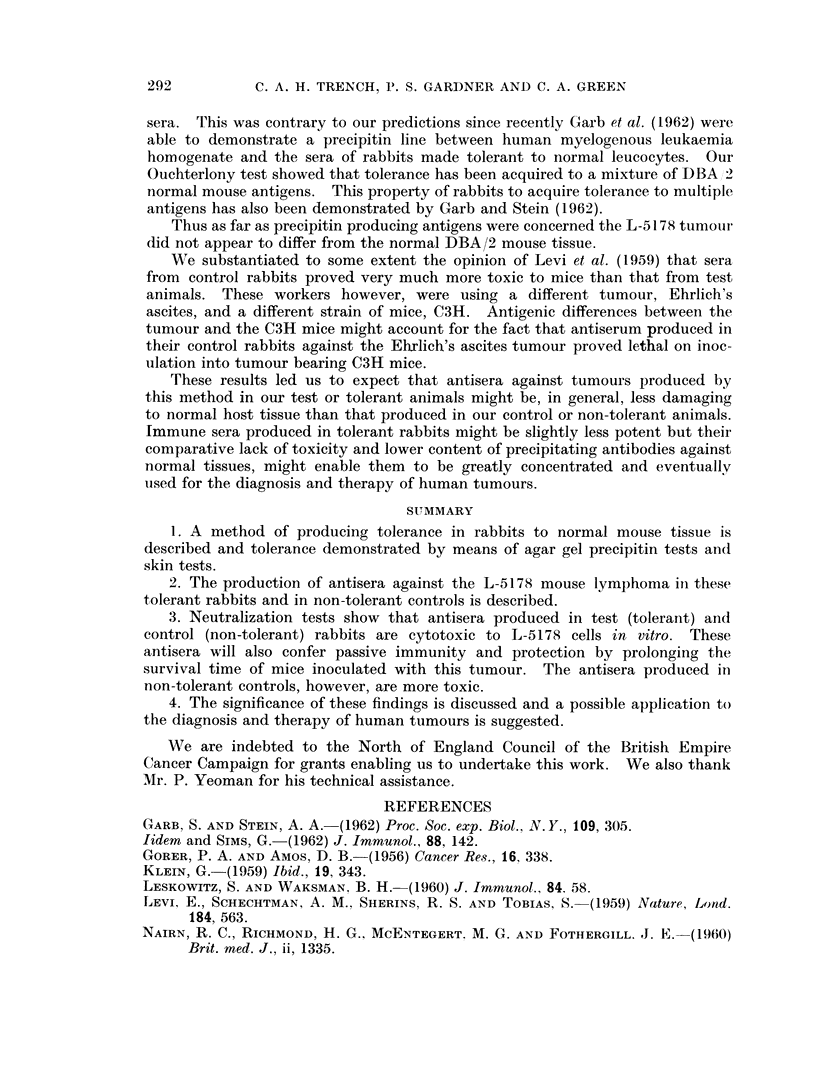# Antibody Production in Tolerant Rabbits to Mouse Lymphoma L-5178

**DOI:** 10.1038/bjc.1963.41

**Published:** 1963-06

**Authors:** C. A. H. Trench, P. S. Gardner, C. A. Green


### ANTIBODY PRODUCTION IN TOLERANT RABBITS TO MOUSE LYMPHOMA L-5178

C. A. H. TRENCH, P. S. GARDNER AND C. A. GREEN

*From the Department of Microbiology, Royal Victoria Infirmary and King's College Medical School, Newcastle Upon Tyne, 1*

Received for publication April 30, 1963

IT was shown by Levi *et al.* (1959) that antiserum to Ehrlich's ascites tumour, produced in rabbits which had been pre-treated during the neonatal period with injections of normal C3H mouse tissue homogenate, was more effective in protecting mice against this tumour than was antiserum produced in normal control rabbits. Working with human myelogenous leukaemia Garb, Stein and Sims (1962) demonstrated that precipitating antibodies against leukaemia cells could be produced in rabbits which had been similarly pre-treated with normal leucocytes.

The following experiment was designed to show whether anti-L-5178 mouse lymphoma serum produced in rabbits pre-treated with normal DBA/2 mouse tissue, would have a cytotoxic effect against this mouse tumour *in vitro* and in addition show the properties of neutralization and protection in mice inoculated with this tumour.

### MATERIALS AND METHODS

*Mice*.—The mouse strain used was the closely inbred DBA/2, which was the strain from which L-5178 tumour originated. These mice were obtained from the Laboratory Animals Centre, Carshalton, and maintained over the past year under their subcultivation scheme. This consisted essentially of mating mice only within their own generation and not keeping the colony for experimental use for more than 3 generations.

*Rabbits*.—Newborn rabbits were divided into 3 groups designated as test, control and normal. Test rabbits were pre-treated in the neonatal stage with normal mouse antigen and later immunized with mouse tumour antigen. Control rabbits received no pre-treatment in the neonatal stage but were later immunized with mouse tumour antigen. Normal rabbits received neither pre-treatment nor immunization and were used solely as a source of normal rabbit serum.

*Mouse lymphoma L*-5178.—This tumour is a lymphocytic neoplasm originating in 1952 in the thymus of a DBA/2 mouse treated with methylcholanthrene at the National Cancer Institute (Carcinogenesis Section) Bethesda, Maryland, U.S.A. (Law, L. W., 1961, personal communication). This cell line was obtained by us from the Chester Beatty Institute of Cancer Research in 1961 and has been maintained in our inbred strain of DBA/2 mice by weekly passage as an ascites cell, by intraperitoneal injections of 0·2 ml. of a 1 in 10 dilution of ascitic fluid in normal saline.

*Normal mouse antigen*.—Four month old DBA/2 mice were killed and the liver, lungs, kidneys, spleen and thymus removed aseptically. These organs were washed in Hanks buffered salt solution containing antibiotics at the following concentrations :—

Penicillin 500 units/ml., streptomycin 250 $\mu$g./ml., neomycin 125 $\mu$g./ml., nystatin 62·5 units/ml. Homogenization in an equal volume of the above solution was carried out in an M.S.E. tissue blender. The homogenate was then further diluted to give a 20 per cent suspension and this was treated with No. 11 Ballotini beads on a Mickle shaker to effect fine disintegration of the tissues. The suspension was finally strained through a copper sieve of average pore size 152 $\mu$ to ensure that the material could be inoculated through a small bore needle. These operations were performed at 4° C. The homogenate was tested for bacteriological sterility and then stored at −20° C.

*Mouse lymphoma L*-5178 *antigen*.—Nineteen 6 month old DBA/2 mice were each inoculated with 0·2 ml. of a 1 in 10 dilution in normal saline of ascitic fluid from a tumour-bearing mouse. On the 8th day the mice were killed, the solid tumour removed from the peritoneal cavity and washed three times in buffered salt solution containing antibiotics as above. The tumour was then homogenized and subsequent treatment was the same as for tissues used for preparation of the normal mouse antigen.

*Adjuvant*.—This consisted of a modification of Freund's formula (Leskowitz and Waksman, 1960) : Bayol, F. 8·5 volumes, Arlacel A. 1·5, volumes, freeze-dried heat killed tubercle bacilli, 20 mg. Equal volumes of this mixture and the tumour antigen were homogenized in the blender and used for immunization.

*Rabbit antisera*.—Blood was obtained from test and control rabbits preceding immunization and on the 14th and 28th day after the last immunizing injection. Sera were stored at −20° C.

*Pre-treatment of test rabbits*.—Within 12 hours of birth the course of inoculations was started. This consisted of 1 ml. of normal mouse antigen intraperitoneally on alternate days for 21 days. Certain precautions were found necessary in handling the litters. The hands were smeared with dilute oil of aniseed and the mother was then removed to another room so that she could not observe the inoculation of her young. A total of 11 rabbits out of 23 survived this treatment.

*Immunization of test and control rabbits*.—At 3 months of age the 11 pre-treated test rabbits together with 15 untreated controls received weekly intraperitoneal inoculations of 1 ml. of tumour antigen for 4 weeks. At the end of the 2nd and 4th weeks, additional antigen (0·4 ml.) together with modified Freund's adjuvant was given intradermally at 4 different sites.

*Skin tests*.—A small area on each flank was shaved. 0·2 ml. of normal mouse antigen was injected intradermally on one side and 0·2 ml. of tumour antigen on the other. The rabbits were challenged at the same sites with the same antigens 10 days later in order to elicit an Arthus reaction. The extent of the reaction was measured in arbitrary units obtained by multiplying the diameter of the indurated area by its thickness. This was found to be maximum at 6 hours.

*Ouchterlony technique*.—5 ml. of 1 per cent Ionagar No. 2 (Oxoid) in distilled water was poured into 2·5 inch petri dishes. When the agar had set, holes were cut using a standard 6 hole cutter (Shandon Scientific Co.) with an additional centre cup. The reagents to be tested were pipetted into the cups and left at room temperature until lines appeared. The plates were then stored at 4° C. The pre-immunization samples of blood, and the 14th and 28th day post-immunization samples were all tested.

*Tumour neutralization test* (IN VITRO).—Tumour cell suspension containing 500,000 cells/ml. in normal saline were incubated at 37° C. for 3 hours with equal volumes of pooled sera from test, control and normal rabbits. Mice, in groups of four, were then given intraperitoneal inoculations of 0·2 ml. of this mixture so that each mouse received 50,000 cells. Survival times of these groups were compared.

*Tumour neutralization tests* (IN VIVO).—A tumour cell suspension consisting of 50,000 cells on 0·2 ml. normal saline was given intraperitoneally to each of 30 mice. The mice were divided into 3 groups and treated with 0·4 ml. of 28th day post-immunization sera from the test, control and normal rabbits. The time interval between inoculation of tumour cells and commencement of serum treatment was varied, intervals of 15 minutes, 18 hours and 36 hours being selected. Once started the injections of sera were repeated every 48 hours until the mice receiving normal rabbit serum had died. Survival times of these groups were compared.

*Mouse protection tests.*—Ten mice were treated with 0·4 ml. of sera from control rabbits on alternate days for 8 days. Six hours after the last injection 500,000 cells in 0·2 ml. normal saline were given intraperitoneally to each mouse.

### RESULTS

*Skin tests.*—Table I shows the reactions measured in arbitrary units of test, control and normal rabbits to normal and tumour antigens.

TABLE I.—*Skin Tests*

| Rabbits | Reaction to normal mouse antigen (units) | Reaction to mouse tumour antigen (units) |
|---|---|---|
| Test | 39 | 26 |
| Control | 120 | 158 |
| Normal | Negative | Negative |

*Ouchterlony tests.*—None of the immune sera from 11 test rabbits showed any precipitin lines to either normal or tumour antigen. All 15 immune sera from the control rabbits showed two or three lines against both antigens.

None of the pre-immunization sera from test or control rabbits showed any precipitin lines against either antigen.

Both normal mouse sera and sera from tumour-bearing mice gave a single diffuse broad band against the test and control rabbit sera. This line was readily distinguishable from the sharp, narrow lines produced by the normal and tumourous mouse antigen against the immune rabbit sera and was probably due to mouse serum proteins.

The general pattern of precipitin lines produced by normal and tumourous mouse tissue against the control rabbit sera indicated that the antigens concerned were similar.

*Tumour neutralization test* (IN VITRO).—Table II shows the survival times of 50 per cent of mice inoculated with tumour cells which had been previously incubated with test, control and normal rabbit sera. Pre-immunization sera from test and control rabbits showed no effect on survival time when compared with normal rabbit sera.

TABLE II.—*Neutralization Tests* (IN VITRO)

| Rabbit sera | 50 per cent survival time of mice |
|---|---|
| Test, 28th day immune sera . | No deaths from tumour |
| Control 28th day immune sera . | No deaths from tumour |
| Normal sera . . . . . | 14 days |

*Tumour neutralization tests* (IN VIVO).—Table III shows the survival times of 50 per cent of tumour-bearing mice which had been inoculated at various times with serum from test, control and normal rabbits. Pre-immunization sera from test and control rabbits showed no effect on survival time as compared with normal rabbit sera.

TABLE III.—*Neutralization Tests* (IN VIVO)

| Rabbit sera used for treatment | Time interval between initiation of treatment and inoculation of tumour | 50 per cent survival time of mice (days) |
|---|---|---|
| Test, 28th day immune sera . | 15 minutes . | 23* |
| Control ,, ,, ,, ,, . | ,, ,, . | 30† |
| Normal sera . | ,, ,, . | 15 |
| Test, 28th day immune sera . | 18 hours . | 16 |
| Control ,, ,, ,, ,, . | ,, ,, . | 17 |
| Normal sera . | ,, ,, . | 13 |
| Test, 14th day immune sera . | 36 hours . | 14 |
| Control ,, ,, ,, ,, . | ,, ,, . | 13 |
| Normal sera . | ,, ,, . | 13 |

* One mouse survived for 3 months with no evidence of tumour at necropsy.
† Four mice alive after 10 months, with no evidence of tumour growth.

*Mouse protection test.*—Table IV shows survival times of 50 per cent mice treated with sera from control and normal rabbits prior to inoculation of tumour.

TABLE IV.—*Mouse Protection Test*

| Rabbit sera | 50 per cent survival time of mice (days) |
|---|---|
| Control, 28th day immune sera . | 16 |
| Normal sera . . . . . | 12 |

*Toxic effects of sera.*—The sera from the control rabbits appeared to produce signs of anaphylactic shock in the mice. This was manifested by shivering, weakness and convulsions lasting for about 30 minutes after the inoculations. In preliminary experiments several mice died within a few hours of administration of the 14th day post-immunization sera. Mice inoculated with sera from the test rabbits exhibited these signs to a noticeably lesser degree, recovering completely in 10–15 minutes ; mice inoculated with normal rabbit sera were unaffected.

*Effect of sera on growth of tumour.*—It was found at post-mortem examination that a plaque of tumour was present at the point of entry of the needle into the peritoneal cavity, in about 80 per cent of the mice inoculated with normal rabbit sera. Infiltrations of this sort were not seen in mice treated with sera from either test or control rabbits.

### DISCUSSION

The purpose of this study was two-fold. In the first place our object was to see whether rabbits could be made wholly or partially tolerant to normal DBA/2 mouse tissue antigens and secondly whether antisera produced in these tolerant rabbits against L-5178 tumour would be as cytotoxic to the tumour as antisera prepared in untreated controls.

The *in vivo* neutralization test showed that, when treatment was started within 15 minutes of the tumour cell inoculation, survival time was prolonged by both control sera and test sera, though less so by the latter. When the interval between initiation of treatment and inoculation of tumour cells was increased to 18 hours, prolongation of survival time was decreased and at 36 hours neither test or control sera produced any significant prolongation of survival time.

Thus it appeared that the test sera, in spite of having been produced in rabbits tolerant or partly tolerant to normal mouse tissue antigens, were almost as potent neutralizing agents as the sera produced in untreated controls.

The *in vitro* neutralization test showed that immune sera from both test and control rabbits had a strong cytotoxic effect on the mouse tumour cells, whereas normal rabbit sera and sera from test and control rabbits before immunization had no cytotoxic effect. The damage to the cells therefore appeared to be caused by circulating antibody, rather than by complement or some other cytotoxic substance present in these sera.

These effects might have been due partly to the action of antibody against normal tissue antigens present in the tumour and partly against a tumour antigen or other antigen not present in normal DBA/2 mouse tissue. The fact that control sera tended to prolong the survival time of the mice to a greater extent than did the test sera was in favour of the former explanation. On the other hand, the fact that the test sera, which were apparently devoid of precipitating antibodies against either normal or tumour antigens were nevertheless capable of prolonging survival time is an indication that it might contain antibody against an antigen not present in normal DBA/2 tissue. This antigen might be a tumour antigen, of the type demonstrated on the EL4 leukosis by Gorer and Amos (1956) or a mutant normal antigen of the type which Klein (1959) has shown might develop as a result of continuous transplantation over a long period. These antigens however, were not demonstrated in the Ouchterlony plate tests.

There appeared to be quite a significant difference in the skin reactions to normal mouse and tumour antigen in the test and control rabbits, the controls responding approximately 4 times as strongly as the test rabbits. This was taken to indicate tolerance to normal tissue antigens and serum proteins, induced by pre-treatment of the test rabbits. Since the reactions were of Arthus type it was considered that the test rabbits have a diminished ability to produce circulating antibody in response to these antigens.

The skin reaction by both test and control rabbits against tumour tissue was less than that against normal tissue. This might be an indication of some loss of antigenicity by the tumour tissue. Lack of organ specific antigens has been demonstrated in the rat liver, hamster kidney and human skin tumours by Nairn *et al.* (1960).

The results of the Ouchterlony tests showed an inability to produce precipitating antibody on the part of the test rabbits. It was unexpected to find that no precipitin lines at all appeared between the normal or tumour antigen and the test sera. This was contrary to our predictions since recently Garb *et al.* (1962) were able to demonstrate a precipitin line between human myelogenous leukaemia homogenate and the sera of rabbits made tolerant to normal leucocytes. Our Ouchterlony test showed that tolerance has been acquired to a mixture of DBA/2 normal mouse antigens. This property of rabbits to acquire tolerance to multiple antigens has also been demonstrated by Garb and Stein (1962).

Thus as far as precipitin producing antigens were concerned the L-5178 tumour did not appear to differ from the normal DBA/2 mouse tissue.

We substantiated to some extent the opinion of Levi *et al.* (1959) that sera from control rabbits proved very much more toxic to mice than that from test animals. These workers however, were using a different tumour, Ehrlich's ascites, and a different strain of mice, C3H. Antigenic differences between the tumour and the C3H mice might account for the fact that antiserum produced in their control rabbits against the Ehrlich's ascites tumour proved lethal on inoculation into tumour bearing C3H mice.

These results led us to expect that antisera against tumours produced by this method in our test or tolerant animals might be, in general, less damaging to normal host tissue than that produced in our control or non-tolerant animals. Immune sera produced in tolerant rabbits might be slightly less potent but their comparative lack of toxicity and lower content of precipitating antibodies against normal tissues, might enable them to be greatly concentrated and eventually used for the diagnosis and therapy of human tumours.

### SUMMARY

1. A method of producing tolerance in rabbits to normal mouse tissue is described and tolerance demonstrated by means of agar gel precipitin tests and skin tests.

2. The production of antisera against the L-5178 mouse lymphoma in these tolerant rabbits and in non-tolerant controls is described.

3. Neutralization tests show that antisera produced in test (tolerant) and control (non-tolerant) rabbits are cytotoxic to L-5178 cells *in vitro*. These antisera will also confer passive immunity and protection by prolonging the survival time of mice inoculated with this tumour. The antisera produced in non-tolerant controls, however, are more toxic.

4. The significance of these findings is discussed and a possible application to the diagnosis and therapy of human tumours is suggested.

We are indebted to the North of England Council of the British Empire Cancer Campaign for grants enabling us to undertake this work. We also thank Mr. P. Yeoman for his technical assistance.

### REFERENCES

GARB, S. AND STEIN, A. A.—(1962) *Proc. Soc. exp. Biol., N.Y.*, **109**, 305.

*Iidem* and SIMS, G.—(1962) *J. Immunol.*, **88**, 142.

GORER, P. A. AND AMOS, D. B.—(1956) *Cancer Res.*, **16**, 338.

KLEIN, G.—(1959) *Ibid.*, **19**, 343.

LESKOWITZ, S. AND WAKSMAN, B. H.—(1960) *J. Immunol.*, **84**, 58.

LEVI, E., SCHECHTMAN, A. M., SHERINS, R. S. AND TOBIAS, S.—(1959) *Nature, Lond.* **184**, 563.

NAIRN, R. C., RICHMOND, H. G., MCENTEGERT, M. G. AND FOTHERGILL, J. E.—(1960) *Brit. med. J.*, ii, 1335.